# Active bystander training beyond the pilot: evaluation across learner groups, cohort sizes, and educational settings

**DOI:** 10.3389/fmed.2026.1984839

**Published:** 2026-10-28

**Authors:** Cindy Chew, Elaine Taylor, Fozia Nazir, Lindsey Pope, Patrick J. O’Dwyer, David Young, Taz Goddard-Fuller

**Affiliations:** 1 Undergraduate School of Medicine, University of Glasgow, Glasgow, United Kingdom; 2 Public Services Delivery Scotland, Glasgow, United Kingdom; 3 Department of Neurology, Queen Elizabeth University Hospital, Glasgow, United Kingdom; 4 School of Health & Wellbeing, University of Glasgow, Glasgow, United Kingdom; 5 Department of Mathematics and Statistics, University of Strathclyde, Strathclyde, United Kingdom; 6 Undergraduate School of Medicine, University of Liverpool, Liverpool, United Kingdom

**Keywords:** active bystander, bullying and harassment, educational intervention, equity, diversity, and inclusion, health profession education, medical education, social justice

## Abstract

**Introduction:**

Active bystander training (ABT) has been cited as a solution to bullying and harassment at the place of work. We have previously shown that a pilot study of ABT sessions in a single institution significantly improved medical students’ knowledge of how to recognise disrespectful behaviour. We evaluated whether a previously developed Active Bystander Training programme could be adapted and delivered in a second medical school, to larger cohorts and to a professionally diverse audience, while retaining measurable immediate educational outcomes.

**Methods:**

Learning and teaching materials were updated and uploaded onto an online platform for easy access. Four facilitators from the pilot study (School A) met with colleagues from a large medical school (School B) to plan and adapt ABT sessions to deliver a condensed, small-group training and to larger groups (final-year medical students). ABT was also delivered to a large, mixed group of learners at a national conference.

**Results:**

In a pilot small group of fourth-year medical students (14 students; 11 paired evaluations) from School B, the mean increase in post-intervention scores was 11.6 (95% CI 7.8–15.3, *p* < 0.001). The median increase in post-intervention scores for the larger cohort final-year medical students (377 students; 64 paired evaluation) was 8.5 (approximate 95% CI 8–8.5, *p* < 0.001). Both showed increases in questionnaire scores, although no formal between-group comparison was undertaken. The mean increase in post-intervention scores for learners attending a national conference (121 delegates; 8 paired evaluations) was 4.75 (95% CI 1.2–8.3, *p* = 0.016).

**Conclusion:**

A previously evaluated ABT programme was successfully adapted for delivery in a second medical school, to a whole-year cohort and to a professionally diverse national audience. These findings support the feasibility of wider delivery while identifying the need for longitudinal evaluation of behavioural and implementation outcomes.

## Introduction

Bullying and harassment at work impact negatively patient outcomes and student learning (1–4) The General Medical Council (GMC) places new responsibilities on doctors to act when witnessing discriminatory behaviour or harassment at work (5). Active bystander training (ABT) has been cited as a potential solution and our early experience appears effective in helping students recognise problematic behaviour and develop confidence in active bystander approaches (6–11).

Much of the existing literature is limited to implementation guidance, resources, or single-institution/pilot studies in small-group settings. With increasing medical school class sizes and expanding curricular content, greater demands are placed on faculty. This severely limits their ability to upskill, develop resources, and deliver teaching with authenticity, while maintaining psychological safety for everyone involved. Evidence describing how established ABT interventions perform when adapted across institutions, learner groups, and delivery formats remains limited. This adds to the barriers to incorporating active bystander training into existing medical school curricula.

We have previously shown that ABT comprising a short recap of learning, watching pre-recorded video vignettes, card games, and immersive simulation significantly improved medical students’ knowledge of how to recognise disrespectful behaviour. That pilot identified several priorities for further evaluation, including delivery to a whole-year cohort, delivery without resource-intensive immersive simulation, implementation in another medical school, and application beyond the original learner group.

The aims of this study were therefore as follows:

1. Evaluate immediate educational outcomes following adaptation and delivery of the previously developed ABT programme in a second medical school.
2. Evaluate delivery of an abbreviated ABT programme to a whole-year medical student cohort and a larger professionally diverse audience.
3. Evaluate the feasibility of developing a shared digital resource to support faculty preparation and wider delivery, and describe early indicators of external interest.

## Methods

Briefly, our previous work involved the following components in each active bystander training session (11):

1. An interactive introduction to recap students’ learning to date. Communication skills including the ABC approach and 5Ds framework of how to be an active bystander (15–30 min) (11–14)
2. Watching pre-recorded video vignettes of interactions in clinical environments and discussing observed behaviours and active bystander approaches (×3 cases)
3. Playing an active bystander-themed card game
4. Immersive simulation (×2 cases)
5. Summary and reflection

### Evaluation tool

Outcomes were assessed using an 11-item questionnaire using a 5-point Likert scale, developed and evaluated in our previous study. Respondents were asked if they could recognise discrimination and harassment; their awareness of reporting policies and procedures; and their skills and likelihood of intervening as an active bystander. The tool demonstrated good test–retest stability, with a mean difference of 0.04 (95% CI −2.36 to 2.45) between repeated baseline administrations approximately 1 week apart. In the present study, it was used without modification across the different learner groups (Supplementary Material 1).

#### Adaptability and delivery across educational settings

Colleagues from two similar-sized medical schools (Schools A and B with ∼400 students per medical year group) worked closely to understand the existing programmatic offering and resource availability within the equity, diversity, and inclusion (EDI) framework of each institution's curriculum. Both institutions reported existing exposure to active bystander training at the start of this study.

Ethical approval was sought and received.

(i) Adaptation and delivery at School B (pilot)

Members of the School A team (from the original study) met with colleagues from School B to plan which components of the ABT session should be included and which might be considered “nice to have.” With scalability and resource limitations in mind, these pilot training sessions to fourth-year medical students were shorter (2 compared with 3 h), excluding the immersive high-fidelity simulation component from the previous format and delivered to slightly larger groups of students (14 compared with a mean of 9). The focus was on understanding the principles of active bystander, working through scenarios, and learning how each of the 5D strategies could be deployed. Due to time constraints, there were reduced opportunities for students to practice active bystander strategies compared to the previous study.

Four members of the original team from School A travelled to School B to deliver the training together with one local host facilitator. This formed part of a planned faculty development and community-of-practice exercise.

Student participation was voluntary. Participants received the same pre-training material and were invited to fill out the same evaluation tool before and after active bystander training. The training and provisions, including availability of a quiet room, were as previously described (11). A statistician guided the number of students needed to adequately power the study.

(ii) Whole Year-5 cohort delivery at School B

Feedback from the pilot session at School B informed the development of content for the roll-out of an Active Bystander session delivered to all Year-5 (final-year) students in the same academic year. Students involved in the pilot told us they would have valued the opportunity to spend time practicing verbalising their bystander responses so that the first time they needed to use them, they would be able to recall appropriate phrases. This led to creation of a version of the session for delivery at scale—a 1.5-h face-to-face workshop, delivered to 16 students at a time, which was formatted as follows:

Section 1: Principles of Active Bystander Activity and the 5D model
Section 2: Putting it into practice: finding the words to respond in challenging situations
Section 3: How to respond as an FY1; discussion of shared video scenarios.

As part of the original ethical approval for the study, permission was obtained to use the same pre- and post-training questions for the roll-out of this version of the workshop with the full cohort. While the Active Bystander activity was a mandatory component of a larger training day, taking part in the evaluation element was voluntary.

(iii) Delivery to a professionally diverse national audience

Members from the original pilot team were invited to deliver an Active Bystander Training workshop to doctors and allied professionals attending a professional conference on Women in Medical Leadership (Royal College of Physicians Edinburgh, June 2025). This provided an opportunity for exploratory evaluation of delivery beyond undergraduate medical education. The ABT workshop was delivered in 1.5 h in a hybrid format and was not recorded to maintain psychological safety. Participants were invited to fill the pre- and post-training evaluation tool used in the previous study anonymously (respondents were prompted to list a colour, shape, or animal to allow pairing of responses).

Institutional ethical board review was sought and waived as responses were anonymous, voluntary, and deemed part of workshop evaluation.

#### Developing digital resource and sharing platform

To support consistent delivery, faculty preparation, and future dissemination, digital resources were developed and deposited on a virtual sharing platform:

(i) Training slides and card game scenarios (*N* = 33) were refreshed.
(ii) A new resource comprising exemplar “what to say/do” responses for active bystanders (“Speak up”) was developed (*N* = 28).
(iii) Video vignettes were reviewed and new material developed (*N* = 12). Colleagues from the Learning Material Development and Academic Services re-recorded the scenarios professionally. These were co-produced with student volunteers, who were remunerated for their time.
(iv) All the resources were uploaded onto a secure, password-protected website with the assistance of a web designer (16 Design Ltd, 16.uk.com).

#### Interest in using the resource

Our experience, results, and resources were shared at conferences, workshops, webinars, and by word of mouth between November 2024 and July 2025. Colleagues were encouraged to get in touch if they were interested in building a community of practice or exploring our resources for their institutional educational use.

### Statistical methods

Differences in questionnaire scores were analysed using paired statistical tests. Differences were tested using the Anderson–Darling test for normality, and Paired t-tests or Wilcoxon tests were applied as appropriate for the distribution of the data. All analyses were performed using Minitab (version 22) at a 5% significance level.

## Results

### Adaptability and delivery at School B

Two training sessions were initially planned (November 2024, March 2025). Due to logistical and student-related factors, only one session involving 14 student volunteers was conducted (November 2024). Eleven (78.6% response rate) paired pre- and post-training evaluation forms were collated. Table 1 and Figure 1 demonstrate the results for this group.

**Table 1:** Pre/post-intervention student scores (*N* = 11).

Post intervention scores	Mean	Std. dev	Median	IQR
Pre-intervention	39.91	5.25	39	37–41
Post-intervention	51.45	4.01	53	47–55

**Figure 1:**
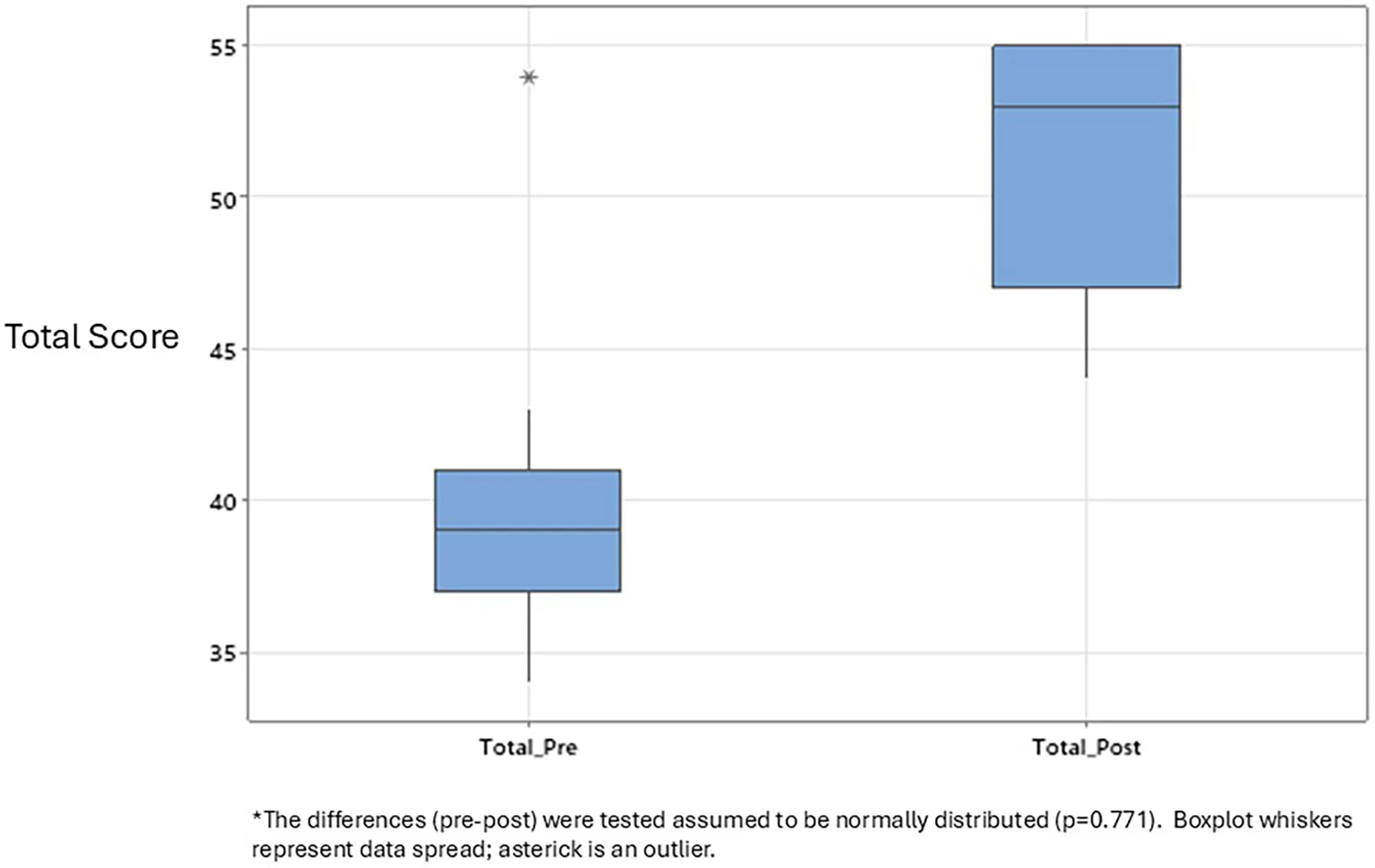
The boxplot shows the distribution of total scores pre- and post-intervention—pilot (paired *N* = 11).

There was evidence of a significant (paired t-test *p* < 0.001) increase in scores post-intervention with a mean increase of 11.6 and 95% CI: 7.8–15.3.

Feedback comments were positive. An exemplar response was “Really enjoyed the discussion and opinions shared. Loved hearing other students’ opinions as well as specific scenario-based advice from the experienced facilitators.”

### Whole Year-5 cohort delivery at School B

ABT was successfully delivered to 377 Year-5 students. Of these, 64 students (17%) provided paired pre/post-evaluation data. Table 2 and Figure 2 demonstrate the results for this group.

**Table 2:** Pre/post-intervention student scores (whole year-5 cohort school B, *N* = 64).

Post intervention scores	Mean	Std. dev	Median	IQR
Pre-intervention	42.17	5.00	42.5	38–46
Post-intervention	50.23	4.32	51	46.25–54.75

**Figure 2:**
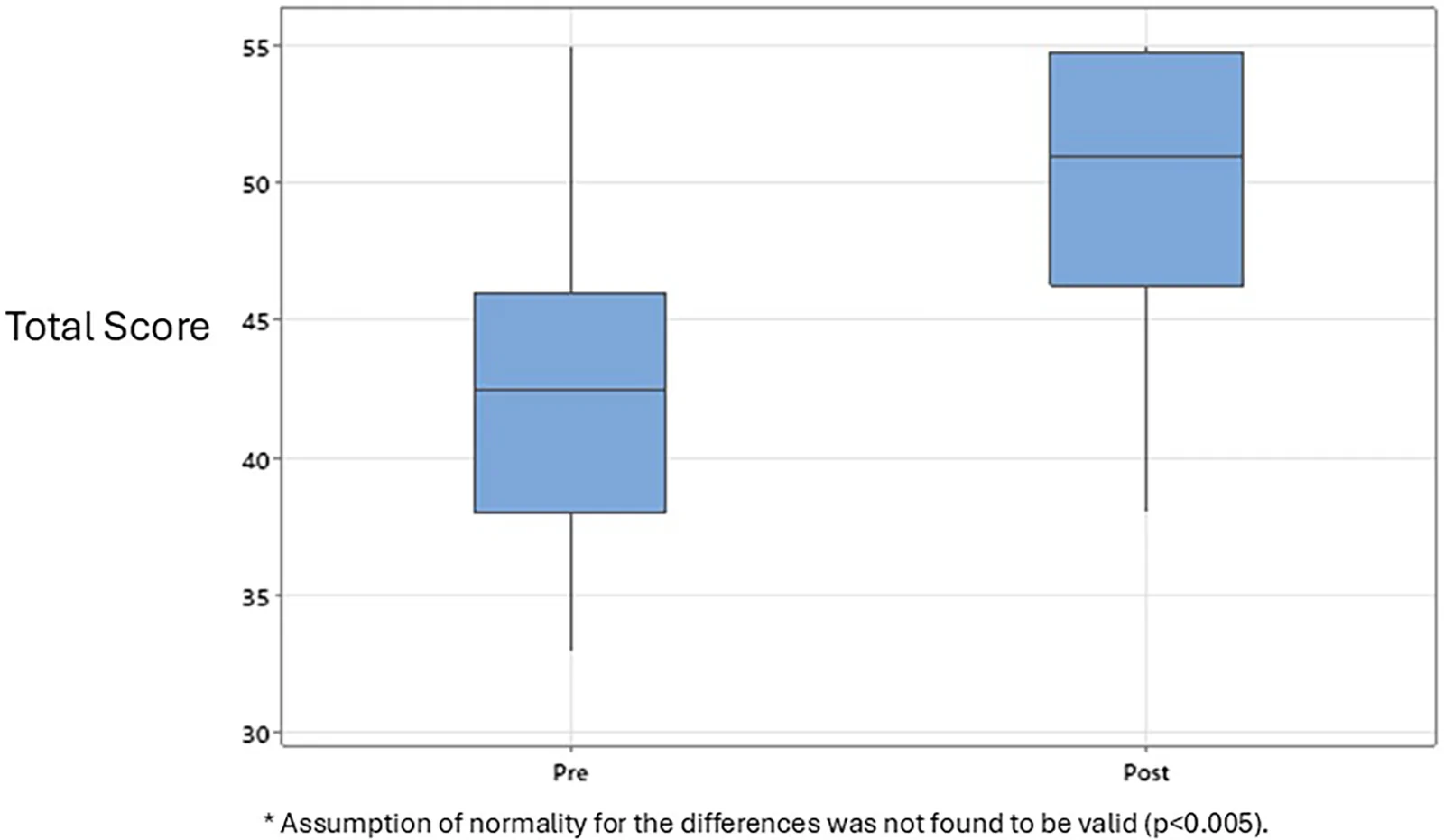
The boxplot shows the distribution of total scores pre- and post-intervention—whole year-5 cohort school B (paired *N* = 64).

There was evidence of a significant (Wilcoxon *p* < 0.001) increase in scores post-intervention with a median increase of 8.5 and approximately 95% CI: 8.0–8.5.

### Delivery to a professionally diverse national audience

One hundred and twenty-one delegates received the intervention, including 110 doctors. The remaining delegates were medical students (*N* = 3) and allied health professionals (AHP).

Three members from the original pilot team (School A) delivered a 1.5-h Active Bystander Training hybrid workshop.

Of the 121 delegates, only 8 (6.6%) participants provided paired pre/post-evaluation data. Table 3 and Figure 3 demonstrate the results for this group.

**Table 3:** Pre/post-intervention scores (professionally diverse national audience, *N* = 8).

Post intervention scores	Mean	Std. dev	Median	IQR
Pre-intervention	41.16	5.44	41.5	35.5–45
Post-intervention	45.88	4.52	44.5	44–49.25

**Figure 3:**
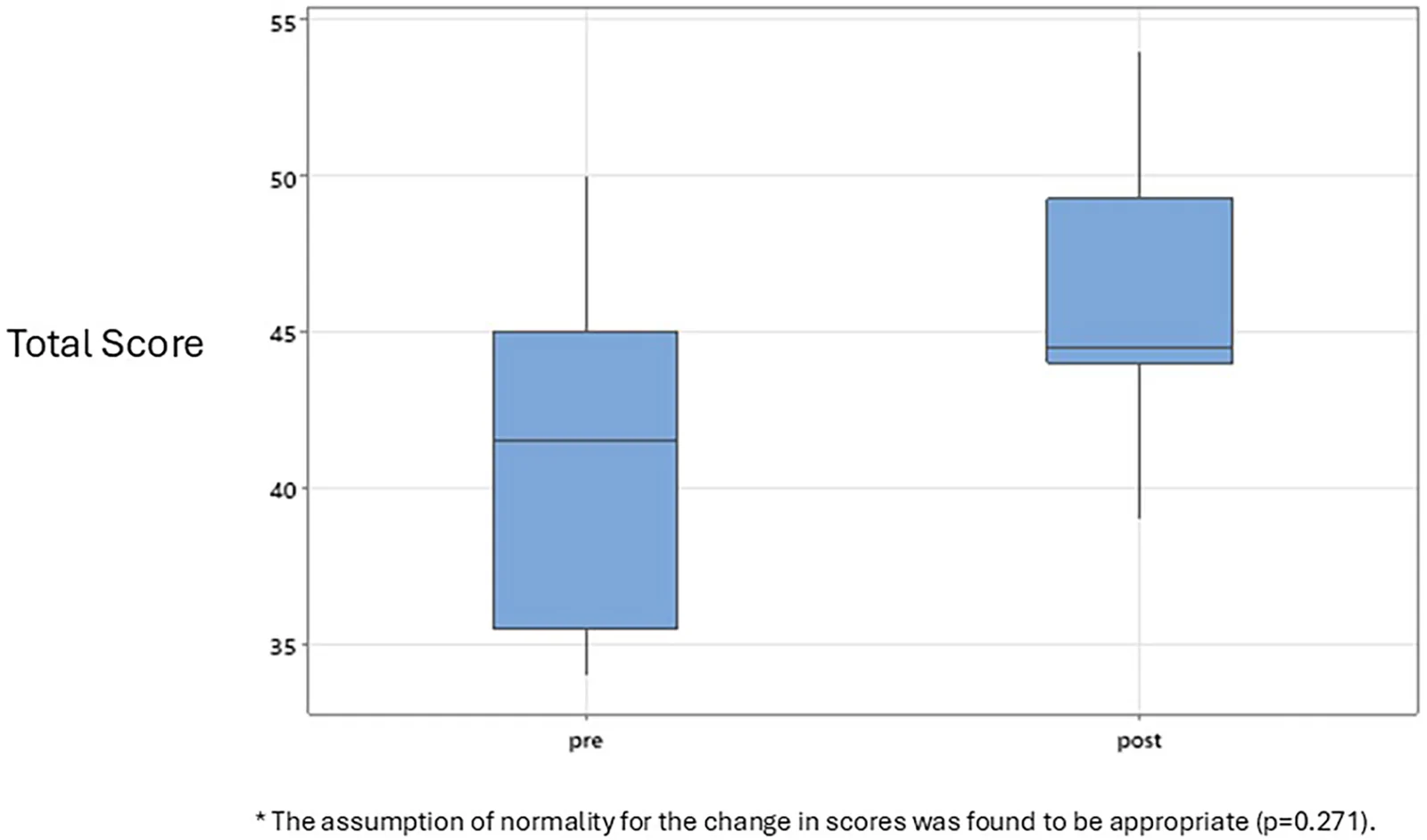
The boxplot shows the distribution of total scores pre- and post-intervention—professionally diverse national audience (paired *N* = 8).

There is evidence of a statistically significant increase in scores post-intervention (paired t-test *p* = 0.016), with a mean increase of 4.75, with 95% CI: 1.2–8.3.

Details of Active Bystander Training adaptations and delivery are summarised in Table 4.

**Table 4:** Adaptation and delivery of active bystander training across settings—summary.

	Original school A^a^	School B pilot	School B whole cohort (Year-5)	National conference
Learners	Medical students	Medical students	Medical students	Doctors/AHP/Medical students
Duration (h)	3	2	1.5	1.5
Delivery	Face-to-face	Face-to-face	Face-to-face	Hybrid
Facilitators (per group)	3	5	2	3
High-fidelity simulation	Yes	No	No	No
Group size	7–12	14	16	121
Total participants	66	14	377	121
Paired evaluations	58	11	64	8
Pre-intervention scores (mean)	38.2	39.9	42.2	41.2
Post-intervention scores (mean)	49.1	51.5	50.2	45.9
Mean change in scores (95% CI; *p*-value)	+11.0 (9.4–12.5; *p* < 0.001)	+11.6 (7.8–15.3; *p* < 0.001)	+8.5^b^ (8.0–8.5; *p* < 0.001)	+4.8 (1.2–8.3; *p* = 0.016)

a Previously published comparator (11).

b Median score as normality for the difference was found not to be valid.

Of the 121 delegates, 54 completed the feedback form. Forty-seven of 54 delegates (87%) rated the workshop 4 or 5 on a 5-point Likert scale (1: poor; 5: excellent). Exemplar feedback comments included “hugely supportive and approachable environment,” “well run workshop,” and “great to have open, supportive and challenging discussions.”

#### Digital resource and sharing platform

Teaching resources were successfully uploaded and hosted on a secure website. Faculty were able to access the resources easily for faculty development, class preparation, and student teaching via the website throughout the period of study.

#### External interest and dissemination

Nine months after the webinars, workshops, conference presentations, and word-of-mouth promotions, colleagues from five educational institutions and hospitals have been in contact to discuss strategies, collaboration, and use of this resource. These contacts represent expressions of interest rather than evidence of adoption or implementation.

## Discussion

This study extends our original pilot by demonstrating that an established ABT intervention could be adapted and delivered in a second medical school, abbreviated from 3 to 1.5 h without high-fidelity simulation, delivered across a whole final-year cohort of 377 students, and delivered to a professionally diverse national audience.

Post-intervention median scores improved by 8.5 (approximate 95% CI 8–8.5) for School B final-year students. This can be compared to a mean improvement of 11 (95% CI 9.4–12.5) in students from School A. The difference in observed change should be interpreted cautiously because both groups differed in baseline scores, learner population, and delivery format, and no formal between-group comparison was undertaken.

Delivery to 121 participants, predominantly doctors, demonstrates feasibility beyond the undergraduate setting. The very low paired evaluation response rate precludes firm conclusions about educational outcomes in this population. Among the eight participants providing paired data, scores increased by 4.75 (95% CI 1.2–8.3; *p* = 0.016); although statistically significant, this finding should be interpreted cautiously given the very small paired sample.

One of the challenges we faced was adequate facilitator training to ensure consistency of the message/content. Balancing active learning and the invitation of shared experience from learners with the potential for adverse responses and cultural (mal)alignment with medical school policy is crucial. Instead of fixed sets of “right”/“wrong” answers, we adopted a “no wrong answer” approach of respectful listening and discussion to promote psychological safety (15). It was therefore critical that faculty were appropriately trained to maintain this atmosphere. However, formal measurement of facilitator fidelity was not undertaken and should form part of future implementation evaluation.

This study demonstrated that a comprehensive set of resources and materials (33 scenarios, 12 video vignettes, and 28 different active bystander strategies) could be successfully adapted to teaching and discussions around equity, diversity, and inclusion beyond its original use. The flexibility of this material is helpful when colleagues are challenged to find the time to develop and deliver essential content to meet GMC requirements. By sharing resources, educators involved in Active Bystander Training can engender further improvements through shared learning.

One of the strengths of this study was embedding co-production with the intended audience throughout the project. Further, the faculty involved were senior educators, bringing lived experiences and a deep understanding of the subject matter, educational ecosystem and curricula. Inter-institutional collaboration, learning, development, and support combined to create the start of a community of practice.

This study has several limitations. The evaluation used an immediate pre- and post-intervention design based on self-reported outcomes and therefore assesses immediate participant responses and perceived changes in knowledge and confidence, rather than observed behavioural change or longer-term sustainability. As the post-intervention scores approached the upper end of the scale (scale range 11–55) in some groups, a ceiling effect cannot be excluded. The low paired response rates, particularly in the whole-cohort and conference group, introduce the possibility of non-response and selection bias, and the direction of this bias cannot be determined from the available data. The smaller School B pilot group and variation in delivery formats and participant populations are further limitations, but they also reflect the pragmatic challenges of implementing and adapting educational interventions within different real-world settings. Accordingly, our findings should be interpreted as evidence of feasibility and consistency of short-term educational outcomes across settings, rather than evidence of sustained behavioural change or system-level scalability.

Whether educational gains following ABT translate into observed bystander behaviour or changes in workplace culture remains unknown. These represent subsequent levels of educational outcome that were beyond the scope of this immediate post-intervention evaluation and are important outcomes for longitudinal evaluation.

## Conclusion

A previously evaluated ABT programme was successfully adapted for delivery in a second medical school, abbreviated for whole-cohort delivery and delivered to a professionally diverse national audience. Significant immediate improvements in questionnaire scores were observed among participants providing paired evaluations across settings, although low response rates in the larger cohorts limit generalisability. These findings support the feasibility of adapting and delivering ABT in different educational contexts; longitudinal and implementation-focused studies are required to evaluate behavioural transfer, fidelity, resource requirements, institutional adoption, sustainability, and organisational outcomes.

## Data Availability

The original contributions presented in the study are included in the article/Supplementary Material, further inquiries can be directed to the corresponding author.
