# Supplementary material for: Active bystander training beyond the pilot: evaluation across learner groups, cohort sizes, and educational settings
Source: Front Med (Lausanne). 2026 Oct 28;13:1984839. doi: 10.3389/fmed.2026.1984839 (PMC13751476; doi:10.3389/fmed.2026.1984839)
Supplement: Supplementary file 1 [file Datasheet1.pdf]

Glasgow Active Bystander Training Questionnaire (#1)

The questionnaire you are about to fill should take just a few moments and will help us improve on the training you are about to receive. This is not a knowledge test and there are no right or wrong answers. Your response will not impact your grades or progression during this clinical block.

Please answer the following questions regarding your confidence and ability to recognise harassment or disrespect on a scale of 1 to 5. (1) indicates no confidence/awareness and (5) indicates maximal confidence/awareness.

Thank you!

Questions Responses Settings

I know the 9 characteristics protected under the UK Equalities Act 2010. *

1	2	3	4	5
○	○	○	○	○

I can identify discrimination based on protected characteristics. *

1	2	3	4	5
○	○	○	○	○

I can identify harassment at the work place. *

1	2	3	4	5
○	○	○	○	○

I feel confident identifying when microaggression occurs at work. *

1	2	3	4	5
○	○	○	○	○

I know when physical contact is inappropriate. *

1	2	3	4	5
○	○	○	○	○

I can identify when someone is being humiliated in public. *

1	2	3	4	5
○	○	○	○	○

I can distinguish constructive feedback from persistent and unjust critical comments/monitoring of my work. *

1	2	3	4	5
○	○	○	○	○

I can identify if someone is under unreasonable pressure to produce work to a challenging standard or deadline. *

1	2	3	4	5
○	○	○	○	○

This next section will gauge your likelihood of intervening as an Active Bystander on a scale of 1 - 5. (1) indicates LOW awareness/likelihood and (5) indicates HIGH awareness/likelihood.

Description (optional)

I am aware of policies and procedures I can follow if I witness harassment or disrespectful behavior. *

1	2	3	4	5
○	○	○	○	○

I have a set of skills I can use to help if I witness harassment or disrespectful behavior. *

1	2	3	4	5
○	○	○	○	○

I feel able to act as a bystander if I witness harassment or disrespectful behavior. *

1	2	3	4	5
○	○	○	○	○

The next question is optional but will help us understand the students undergoing this training.

Description (optional)

Do you identify as having a protected characteristic under the Equalities Act?

- ○ Yes
- ○ No
- ○ Prefer not to say
